# Real-world effectiveness after initiating fremanezumab treatment in US patients with episodic and chronic migraine or difficult-to-treat migraine

**DOI:** 10.1186/s10194-022-01415-x

**Published:** 2022-05-16

**Authors:** Maurice T. Driessen, Joshua M. Cohen, Stephen F. Thompson, Oscar Patterson-Lomba, Michael J. Seminerio, Karen Carr, Todor I. Totev, Rochelle Sun, Erica Yim, Fan Mu, Rajeev Ayyagari

**Affiliations:** 1 Teva Pharmaceuticals, Piet Heinkade 107, 1019 BR Amsterdam, The Netherlands; 2 Teva Branded Pharmaceutical Products R&D, Inc., West Chester, PA USA; 3 Teva Pharmaceuticals, Parsippany, NJ USA; 4 Analysis Group, Boston, MA USA

**Keywords:** Migraine, Fremanezumab, CGRP, Migraine preventive treatment, Real-world effectiveness, Chart review, Difficult-to-treat

## Abstract

**Background:**

Fremanezumab, a fully humanized monoclonal antibody (mAb; IgG2Δa) that selectively targets calcitonin gene-related peptide (CGRP), is approved for the preventive treatment of migraine in adults. The efficacy and safety of fremanezumab for migraine prevention have been demonstrated in randomized, double-blind, placebo-controlled trials. Real-world effectiveness data are needed to complement clinical trial data. This study assessed the effectiveness of fremanezumab across different subgroups of adult patients with episodic migraine (EM), chronic migraine (CM), or difficult-to-treat (DTT) migraine in real-world clinical settings.

**Methods:**

This retrospective, panel-based online chart review used electronic case report forms. Patient inclusion criteria were a physician diagnosis of EM or CM; age ≥ 18 years at the time of first fremanezumab initiation; ≥ 1 dose of fremanezumab treatment; ≥ 1 follow-up visit since first initiation; and ≥ 2 measurements of monthly migraine days (MMD; with 1 within a month before or at first initiation and ≥ 1 after first initiation). Changes in MMD and monthly headache days were assessed during the follow-up period. These endpoints were evaluated in subgroups of patients by migraine type (EM/CM) and in subgroups with DTT migraine (diagnosis of medication overuse [MO], major depressive disorder [MDD], generalized anxiety disorder [GAD], or prior exposure to a different CGRP pathway–targeted mAb [CGRP mAb]).

**Results:**

Data were collected from 421 clinicians and 1003 patients. Mean (percent) reductions from baseline in MMD at Month 6 were − 7.7 (77.0%) in EM patients, − 10.1 (68.7%) in CM patients, − 10.8 (80.6%) in the MO subgroup, − 9.9 (68.3%) in the MDD subgroup, − 9.5 (66.4%) in the GAD subgroup, and − 9.0 (68.7%) in the prior CGRP mAb exposure subgroup. Improvements in MDD or GAD severity were reported by 45.5% and 45.8% of patients with comorbid MDD or GAD, respectively.

**Conclusions:**

In this real-world study, fremanezumab demonstrated effectiveness for migraine regardless of migraine type or the presence of factors contributing to DTT migraine (MO, GAD, MDD, or prior exposure to a different CGRP mAb).

**Supplementary Information:**

The online version contains supplementary material available at 10.1186/s10194-022-01415-x.

## Introduction

Migraine is a prevalent, debilitating neurologic disease. In the United States alone, approximately 1 in 6 individuals suffer from migraine [1]. Migraine is the second leading cause of years lived with disability worldwide [2] and the leading cause of years lived with disability in individuals < 50 years of age [3]. Patients with migraine experience significantly reduced health-related quality of life [4, 5] and work productivity [6]. Migraine results in > 100 million lost workdays per year and is associated with > $75 billion in direct and indirect costs per year [7]. Within the migraine population, there are subgroups of patients who may have more difficult-to-treat (DTT) migraine based on prior migraine preventive treatment failure, acute medication overuse (MO), or the presence of psychiatric comorbidities, such as depression or anxiety. Patients with DTT migraine may experience more frequent headache days, more severe headache pain, increased disability, worse quality of life, and an increased risk for migraine chronification [8–12], resulting in increased health care resource utilization and cost burdens associated with migraine [13].

Fremanezumab is a fully humanized monoclonal antibody (mAb; IgG2Δa) that selectively targets the calcitonin gene-related peptide (CGRP) ligand and is approved for the preventive treatment of migraine in adults [14]. Phase 3 randomized clinical studies up to 15 months have demonstrated the long-term efficacy and safety of quarterly and monthly fremanezumab in adults with episodic migraine (EM; 6–14 headache days per month) and chronic migraine (CM; ≥ 15 headache days per month), even those with DTT migraine [15–20].

To expand on the efficacy and safety data generated in phase 3 controlled clinical trial settings [15–20] and following the approval of fremanezumab in 2018 by the US Food and Drug Administration [14], real-world evidence is an important addition to confirm clinical effectiveness and adherence for fremanezumab treatment in patients with migraine, including those with DTT migraine. Therefore, in the current chart review, we evaluated the effectiveness of fremanezumab by migraine type (EM/CM) and among different subgroups of patients with potentially DTT migraine.

## Methods

### Study design

This US-based study was a non-interventional, retrospective, online, clinician panel–based chart review. The study design has been presented elsewhere and is briefly summarized here. Participating clinicians were provided a custom-designed electronic case report form (eCRF) to enter data abstracted from eligible patient charts. The eCRF was used to collect de-identified patient information, including demographic and clinical characteristics, prior treatment patterns, baseline migraine assessments, effectiveness outcomes during follow-up, and incidence of psychiatric comorbidities, for up to 5 patients meeting the inclusion criteria. Participating clinicians were asked to randomly select these patients’ charts using a randomization scheme that was included as part of the underlying program for the eCRF and was based on a randomized sequence of letters. Clinicians were asked to choose a patient chart with the last name corresponding to the random letter produced by the program; the program repeated this randomization process for each additional patient chart selected.

The date of first fremanezumab treatment initiation was designated as the index date.

### Clinician and patient eligibility criteria

Clinicians were selected to participate if they were a neurologist, general practitioner, pain management specialist, psychiatrist, physician assistant (PA), nurse practitioner (NP), or other headache specialist who routinely treated patients with EM or CM in a US-based practice and had treated ≥ 5 patients diagnosed with EM or CM in the past 12 months, including patients who met inclusion criteria. Eligible patients had a physician diagnosis of EM or CM; first initiation with fremanezumab treatment after diagnosis of EM or CM; age ≥ 18 years at the time of fremanezumab initiation; treatment with ≥ 1 dose of fremanezumab; ≥ 1 follow-up visit since first fremanezumab treatment initiation; and chart containing ≥ 2 measurements of monthly migraine days (MMD), 1 prior to first initiation of fremanezumab treatment (pre-index period) or at the index date and ≥ 1 during the follow-up period as an outcome assessment. Patients who were pregnant in the 12 months prior to first initiation or during fremanezumab treatment were excluded.

### Outcome assessment

Patient outcomes were evaluated in subgroups of patients with physician-reported EM or CM, as well as in subgroups of patients with DTT migraine, including patients with physician-reported MO, major depressive disorder (MDD), or generalized anxiety disorder (GAD) and patients with prior exposure to a different CGRP pathway–targeted mAb in the 12 months before first initiation of fremanezumab treatment. Although these subgroups were based on physician report, the eCRF provided physicians with the following definitions as a guide: EM, 0 to 14 monthly headache days (MHD); CM, ≥ 15 MHD; MO, ≥ 10 days per month of use of ergots, triptans, opioids, combination analgesics, or combinations of drugs of different classes not individually overused or ≥ 15 days per month of use of non-opioid analgesics, acetaminophen, or non-steroidal anti-inflammatory drugs. A definition of MDD or GAD was not provided but was based on diagnosis noted in the chart. Baseline MMD and MHD were collected for 1 month pre-index, baseline MDD and GAD severity were collected for 3 months pre-index, and comorbidities and prior treatments were collected for 12 months pre-index. Study outcomes were evaluated during the full follow-up period after first fremanezumab treatment initiation until treatment discontinuation or chart abstraction (i.e., post-index). Effectiveness outcomes that were evaluated during the follow-up period included change from baseline in MMD and change from baseline in MHD at each monthly time point post-index. The proportion of patients with a ≥ 50% reduction in MMD from baseline was also assessed at each monthly time point. For patients with MDD and GAD, respectively, MDD and GAD severity (no symptoms, mild, moderate, or severe) at baseline and the change in MDD and GAD severity (improved, no change, or worsened) post-index were assessed. Clinicians were asked to estimate the percentage of time their patients were adherent to treatment during the full period of the first treatment with a given dosage of fremanezumab. Rates of treatment adherence to fremanezumab dosing schedules were estimated as the percentage of patients complying with treatment administration schedules > 80% of the time, based on those clinician assessments. Adherence, discontinuation rates, and reasons for discontinuation were reported over the full follow-up period, the duration of which varied among patients.

### Statistical analysis

The study included 1003 patients, and from that total population, patients were further selected for these subgroup analyses by migraine type or presence of DTT migraine. For all subgroups, sample sizes were not based on any statistical considerations. Baseline patient demographics, disease characteristics, and treatment characteristics (including concomitant medication use) were summarized using mean (standard deviation [SD]) values for continuous variables and frequency distributions for categorical variables. For effectiveness outcomes during the follow-up period, continuous variables were summarized using descriptive statistics (mean and SD) and categorical variables were summarized using frequency distributions. For these effectiveness outcomes, results were clustered for patients with available assessments within ± 15 days of each reported time point (e.g., Month 1, Month 2) due to heterogeneity in the assessment times observed in real-world practice. As a result, not all patients were included in the analyses for each time point and sample sizes varied accordingly.

## Results

### Clinician and patient characteristics

Of the 421 clinicians included in this study, 240 (57.0%) were neurologists, 80 (19.0%) were general practitioners, 36 (8.6%) were pain management specialists, 21 (5.0%) were psychiatrists, 38 (9.0%) were PAs or NPs, and 6 (1.4%) were other headache specialists. Clinicians had treated a mean (SD) of 367.0 (457.6) patients with migraine in the 12 months prior to the study, including 68.1 (159.2) patients treated with fremanezumab. A total of 1003 patients were included in this study, with these patients initiating fremanezumab treatment between October 2, 2018, and July 17, 2020. Of the 1003 patients included in the study, 416 (41.5%) and 587 (58.5%) patients were diagnosed with EM and CM, respectively; 220 (21.9%) patients had physician-reported MO. A total of 134 (13.4%) patients had physician-reported MDD and 120 (12.0%) patients had physician-reported GAD. Ninety-eight (9.8%) patients had been previously treated with another CGRP pathway–targeted mAb. A total of 622 (62.0%) patients were receiving monthly fremanezumab and 381 (38.0%) were receiving quarterly fremanezumab at treatment initiation. For patients taking monthly versus quarterly fremanezumab, the time from diagnosis to index date was longer (mean [SD], 7.4 [8.8] vs 5.4 [7.9] years), a higher proportion had CM (61.6% [383/1003] vs 53.5% [204/1003]), and the number of MMD and MHD at baseline was higher (mean [SD] MMD, 13.2 [6.5] vs 11.9 [6.1]; mean [SD] MHD, 14.8 [8.0] vs 12.9 [7.7]).

Baseline and demographic characteristics for the subgroups of patients with DTT migraine are summarized in Table 1. The mean (SD) age ranged from 38.5 (12.2) years in the EM subgroup to 43.0 (12.2) years in the prior CGRP pathway–targeted mAb subgroup. The mean (SD) duration of follow-up ranged from 7.0 (4.5) months in the CM subgroup to 7.6 (4.5) months in the GAD subgroup. In the prior CGRP pathway–targeted mAb subgroup, 41% of patients had failed ≥ 5 prior migraine preventive treatments, while only 12% to 29% of patients in the other subgroups had failed ≥ 5 prior preventive treatments (Table 1). Acute and preventive medications taken prior to and concomitantly with fremanezumab treatment are summarized in Supplemental Table 1; both acute and preventive medication use decreased after fremanezumab initiation.

**Table 1:** Demographic and Baseline Characteristics Across Patient Subgroups

Characteristic	Patients with EM (***n*** = 416)	Patients with CM (***n*** = 587)	Patients with MO (***n*** = 220)	Patients with MDD (***n*** = 134)	Patients with GAD (***n*** = 120)	Prior CGRP exposure (***n*** = 98)
Age, years, mean (SD)	38.5 (12.2)	40.5 (12.5)	40.7 (12.4)	40.1 (10.6)	39.0 (11.7)	43.0 (12.2)
Sex, *n* (%)						
Female	301 (72.4)	459 (78.2)	155 (70.5)	110 (82.1)	99 (82.5)	77 (78.6)
Male	115 (27.6)	126 (21.5)	65 (29.5)	24 (17.9)	21 (17.5)	21 (21.4)
Other	0 (0.0)	2 (0.3)	0 (0.0)	0 (0.0)	0 (0.0)	0 (0.0)
Race, *n* (%)						
White	317 (76.2)	465 (79.2)	158 (71.8)	112 (83.6)	99 (82.5)	78 (79.6)
Black or African American	59 (14.2)	78 (13.3)	42 (19.1)	14 (10.4)	11 (9.2)	11 (11.2)
Asian	26 (6.3)	30 (5.1)	10 (4.5)	5 (3.7)	5 (4.2)	1 (1.0)
Native American or American Indian	6 (1.4)	1 (0.2)	2 (0.9)	0 (0.0)	0 (0.0)	1 (1.0)
Other	8 (1.9)	12 (2.0)	7 (3.2)	3 (2.2)	5 (4.2)	6 (6.1)
Unknown	0 (0.0)	1 (0.2)	1 (0.5)	0 (0.0)	0 (0.0)	1 (1.0)
Duration of follow-up, months, mean (SD)	7.1 (4.4)	7.0 (4.5)	7.1 (3.7)	7.3 (4.4)	7.6 (4.5)	7.2 (4.7)
Baseline MMD, mean (SD)	10.0 (5.1)	14.7 (6.5)	13.4 (6.7)	14.5 (6.1)	14.3 (6.3)	13.1 (6.2)
Baseline MHD, mean (SD)	10.7 (6.1)	16.4 (8.3)	15.8 (8.8)	17.5 (8.0)	16.7 (8.3)	14.6 (7.9)
Prior preventive treatment failures, *n* (%)						
0	34 (8.2)	26 (4.4)	1 (0.5)	4 (3.0)	3 (2.5)	1 (1.0)
1	54 (13.0)	57 (9.7)	12 (5.5)	11 (8.2)	9 (7.5)	9 (9.2)
2	120 (28.8)	126 (21.5)	36 (16.4)	20 (14.9)	18 (15.0)	17 (17.3)
3	105 (25.2)	177 (30.2)	62 (28.2)	42 (31.3)	39 (32.5)	17 (17.3)
4	52 (12.5)	109 (18.6)	45 (20.5)	22 (16.4)	20 (16.7)	14 (14.3)
5	18 (4.3)	38 (6.5)	26 (11.8)	12 (9.0)	10 (8.3)	12 (12.2)
≥ 6	33 (7.9)	54 (9.2)	38 (17.3)	23 (17.2)	21 (17.5)	28 (28.6)
Common baseline comorbid conditions, *n* (%)^a^						
Insomnia	64 (15.4)	132 (22.5)	65 (29.5)	49 (36.6)	42 (35.0)	27 (27.6)
Allergies	63 (15.1)	95 (16.2)	38 (17.3)	30 (22.4)	24 (20.0)	19 (19.4)
Neck pain	60 (14.4)	95 (16.2)	51 (23.2)	26 (19.4)	29 (24.2)	28 (28.6)
Hypertension	61 (14.7)	82 (14.0)	32 (14.5)	26 (19.4)	22 (18.3)	18 (18.4)
Back pain	46 (11.1)	92 (15.7)	35 (15.9)	20 (14.9)	24 (20.0)	20 (20.4)
Obesity	47 (11.3)	90 (15.3)	38 (17.3)	37 (27.6)	27 (22.5)	20 (20.4)
MDD	41 (9.9)	93 (15.8)	47 (21.4)	134 (100.0)	39 (32.5)	22 (22.4)
GAD	27 (6.5)	93 (15.8)	52 (23.6)	39 (29.1)	120 (100.0)	22 (22.4)
Chronic pain	32 (7.7)	79 (13.5)	41 (18.6)	26 (19.4)	34 (28.3)	22 (22.4)
Asthma	38 (9.1)	63 (10.7)	33 (15.0)	18 (13.4)	20 (16.7)	13 (13.3)
Clinician-estimated adherence rate, mean (SD)	92.3 (17.6)	95.1 (13.1)	89.8 (19.7)	95.0 (12.3)	95.6 (10.0)	95.0 (11.2)
Patients with ≥ 80% adherence, *n* (%)	362 (87.0)	550 (93.7)	182 (82.7)	125 (93.3)	114 (95.0)	90 (91.8)
Discontinuation rate after fremanezumab index dose, *n* (%)	31 (7.5)	47 (8.0)	17 (7.7)	5 (3.7)	14 (11.7)	12 (12.2)

*CGRP*, calcitonin gene-related peptide; *CM*, chronic migraine; *EM*, episodic migraine; *GAD*, generalized anxiety disorder; *MDD*, major depressive disorder; *MHD*, monthly headache days; *MMD*, monthly migraine days; *MO*, medication overuse; *SD*, standard deviation

^a^Incidence ≥ 10% in the overall study population

### Fremanezumab treatment adherence and discontinuations during the post-index period

Across the EM, the CM, and all DTT subgroups, adherence to fremanezumab treatment was high and fremanezumab treatment discontinuation rates were low after the index dose (Table 1). The majority of patients in all subgroups (≥ 85%) continued on their originally prescribed dosing regimen (monthly or quarterly) of fremanezumab.

### Monthly migraine days and other key outcomes

#### Migraine type subgroups

##### EM subgroup

**Fig. 1:**
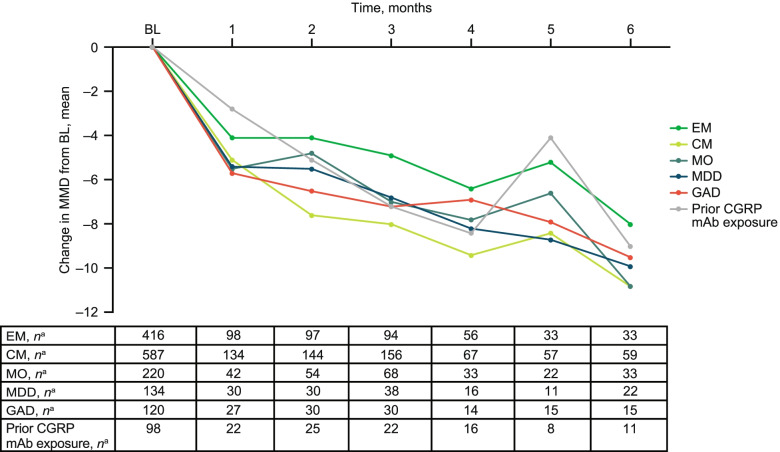
Change from baseline in MMD across patient subgroups. BL, baseline; CGRP, calcitonin gene-related peptide; CM, chronic migraine; EM, episodic migraine; GAD, generalized anxiety disorder; mAb, monoclonal antibody; MDD, major depressive disorder; MMD, monthly migraine days; MO, medication overuse. ^a^Number of patients with available assessment at each time point

**Fig. 2:**
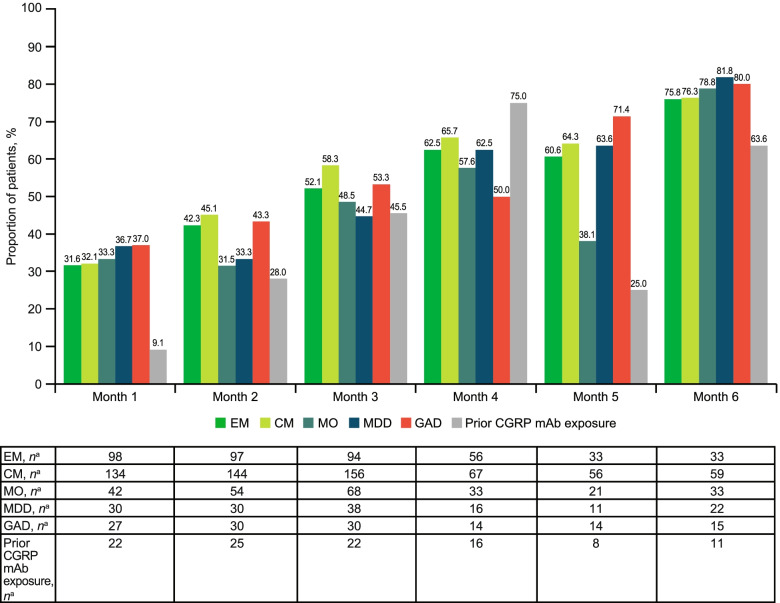
Proportion of patients with ≥ 50% reduction from baseline in MMD across patient subgroups. CGRP, calcitonin gene-related peptide; CM, chronic migraine; EM, episodic migraine; GAD, generalized anxiety disorder; mAb, monoclonal antibody; MDD, major depressive disorder; MMD, monthly migraine days; MO, medication overuse. ^a^Number of patients with available assessment at each time point

Among patients with EM (*n* = 416), the mean (SD) number of MMD at baseline was 10.0 (5.1). Mean (percent) reductions from baseline in MMD were − 3.4 (34.0%) at Month 1, − 4.7 (47.0%) at Month 3, and − 7.7 (77.0%) at Month 6 (Fig. 1). The proportion of patients with ≥ 50% reduction in MMD increased from Month 1 (31.6% [31/97]) to Month 3 (52.1% [49/94]) and Month 6 (75.8% [25/33]; Fig. 2).

##### CM subgroup

In patients with CM (*n* = 587), the mean (SD) number of MMD at baseline was 14.7 (6.5). Mean (percent) reductions from baseline in MMD were − 5.5 (37.4%) at Month 1, − 7.9 (53.7%) at Month 3, and − 10.1 (68.7%) at Month 6 (Fig. 1). The proportion of patients with ≥ 50% reduction in MMD increased from Month 1 (32.1% [43/134]) to Month 3 (58.3% [91/156]) and Month 6 (76.3% [45/59]; Fig. 2). Increasing reductions in MMD over the 6-month treatment period were observed regardless of migraine type (Figs. 1 and 2).

#### MO subgroup

In the MO subgroup (*n* = 220), the mean (SD) number of MMD at baseline was 13.4 (6.7). The mean (percent) reduction from baseline in MMD increased from − 5.5 (41.0%) at Month 1 to − 7.0 (52.2%) at Month 3 and − 10.8 (80.6%) at Month 6 (Fig. 1). The proportion of patients with MO with a ≥ 50% reduction in MMD also increased from Month 1 (33.3% [14/42]) to Month 3 (48.5% [33/68]) and Month 6 (78.8% [26/33]; Fig. 2).

#### MDD and GAD subgroups

##### MDD subgroup

In the MDD subgroup (*n* = 134), the mean (SD) number of MMD at baseline was 14.5 (6.1). Mean (percent) reductions from baseline in MMD were − 5.4 (37.2%) at Month 1, − 6.8 (46.9%) at Month 3, and − 9.9 (68.3%) at Month 6 (Fig. 1). The proportion of patients with a ≥ 50% reduction in MMD increased from Month 1 through Months 3 and 6 (Month 1, 36.7% [11/30]; Month 3, 44.7% [17/38]; Month 6, 81.8% [18/22]; Fig. 2).

**Fig. 3:**
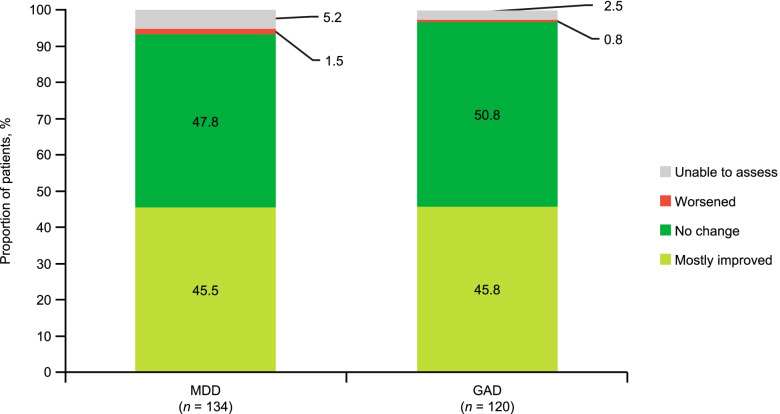
Change in MDD and GAD severity. GAD, generalized anxiety disorder; MDD, major depressive disorder

In the MDD subgroup, the severity of MDD at baseline was reported as no symptoms for 4.5% (6/134) of patients, mild for 37.3% (50/134), moderate for 45.5% (61/134), and severe for 10.4% (14/134). MDD severity was reported as improved by 45.5% (61/134) of patients post-index (Fig. 3).

##### GAD subgroup

In the GAD subgroup (*n* = 120), the mean (SD) number of MMD at baseline was 14.3 (6.3). Mean (percent) reductions from baseline in MMD were − 5.7 (39.9%) at Month 1, − 7.2 (50.3%) at Month 3, and − 9.5 (66.4%) at Month 6 (Fig. 1). The proportion of patients with GAD with a ≥ 50% reduction in MMD increased from Month 1 (37.0% [10/27]) to Month 3 (53.3% [16/30]) and Month 6 (80.0% [12/15]; Fig. 2).

In this subgroup, the severity of GAD at baseline was reported as no symptoms for 1.7% (2/120) of patients, mild for 35.8% (43/120); moderate for 45.8% (55/120), and severe for 15.8% (19/120). GAD severity was reported as improved by 45.8% (55/120) of patients with GAD post-index (Fig. 3).

#### Prior CGRP pathway–targeted mAb exposure subgroup

Among patients who switched from another prior CGRP pathway–targeted mAb to fremanezumab (*n* = 98), 92 (93.9%) provided a reason for switching to fremanezumab. The most common reasons were inadequate response to treatment (defined as no clinically meaningful improvement after ≥ 3 months of therapy; 67.4% [62/92]) and side effects (32.6% [30/92]). Other frequent reasons for switching to fremanezumab included insurance coverage (19.6% [18/92]), the option of quarterly dosing (16.3% [15/92]), availability of free samples (15.2% [14/92]), the long-acting profile of fremanezumab (14.1% [13/92]), preference indicated by the patient (14.1% [13/92]), wearing off prior to the next injection (10.9% [10/92]), and options of both quarterly and monthly dosing (10.9% [10/92]). Many patients indicated > 1 reason for switching.

In the overall subgroup of patients with prior exposure to another CGRP pathway–targeted mAb, the mean (SD) number of MMD at baseline was 13.1 (6.2). Mean (percent) reductions from baseline in MMD were − 2.8 (21.4%) at Month 1, − 7.2 (55.0%) at Month 3, and − 9.0 (68.7%) at Month 6 (Fig. 1). The proportion of patients in the prior CGRP pathway–targeted mAb subgroup with ≥ 50% reduction in MMD increased from Month 1 (9.1% [2/22]) to Month 3 (45.5% [10/22]) and Month 6 (63.6% [7/11]; Fig. 2).

### Monthly headache days

#### Migraine type, MO, MDD, GAD, and prior CGRP mAb exposure subgroups

**Fig. 4:**
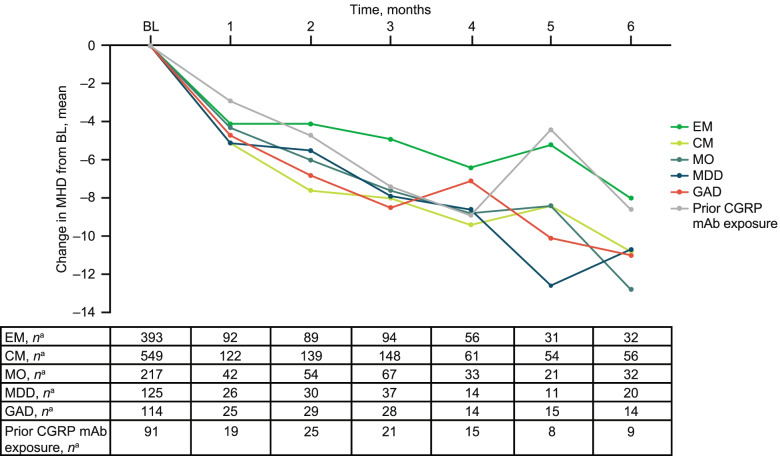
Change from baseline in MHD across patient subgroups. BL, baseline; CGRP, calcitonin gene-related peptide; CM, chronic migraine; EM, episodic migraine; GAD, generalized anxiety disorder; mAb, monoclonal antibody; MDD, major depressive disorder; MHD, monthly headache days; MO, medication overuse. ^a^Number of patients with available assessment at each time point

Mean (SD) numbers of MHD at baseline across all evaluated subgroups are shown in Table 1. Across all subgroups, trends in mean (percent) reductions in MHD were comparable to those shown for MMD (Fig. 4).

## Discussion

There is limited real-world evidence on the use of fremanezumab for the preventive treatment of migraine, especially among patients with DTT migraine. The current clinician panel–based retrospective chart review study provides a timely and comprehensive assessment of the effectiveness of fremanezumab in a real-world setting across a variety of patient subgroups, including those with potentially DTT migraine. MMD and MHD reductions observed in the current subgroup analyses supported the effectiveness in patients with EM or CM and those with DTT migraine, based on the presence of MO, GAD, MDD, or prior CGRP pathway–targeted mAb exposure.

Regardless of the type of migraine (EM or CM), reductions from baseline in MMD and MHD were observed within 1 month after initiating fremanezumab treatment, with continued improvement demonstrated over 6 months of treatment. The proportion of patients with ≥ 50% reduction in MMD also increased from Month 1 to Month 6 of fremanezumab treatment in both the EM and CM subgroups, and discontinuation rates were low regardless of the subgroup studied. These real-world effectiveness results complement those from the randomized, double-blind, placebo-controlled, phase 3 HALO studies, which reported significant reductions in MMD with fremanezumab versus placebo over 3 months of treatment in patients with EM or CM [15, 16]. Likewise, over the course of a 12-month extension study, the proportion of patients with ≥ 50% reduction in MMD also increased over time and discontinuation rates were low [20]. In the randomized, double-blind, placebo-controlled, phase 3b FOCUS study, which included a similar population to the current real-world study (patients with EM or CM with inadequate response to 2 to 4 prior classes of migraine preventive treatments), patients who received fremanezumab had significant reductions in MMD compared with those assigned to placebo over 3 months of treatment and the proportion of patients with ≥ 50% reduction in MMD increased over an additional 3-month open-label extension [17, 21]. Compared with phase 3 trials [15–17], which showed least-squares mean changes from baseline in MMD ranging from − 3.4 to − 5.0 over 3 months for patients with EM or CM, the current study demonstrated even larger mean changes from baseline in MMD at 3 months in patients with EM or CM (− 4.7 to − 7.9), highlighting the effectiveness of fremanezumab in the real-world setting. These differences in real-world and clinical trial outcomes are in line with those observed for other CGRP pathway–targeted mAbs erenumab and galcanezumab [22–28].

The current study also demonstrated reductions in MMD and MHD with fremanezumab treatment for patients diagnosed with MO. These real-world outcomes support the findings of previous subgroup analyses of the randomized, double-blind, placebo-controlled, phase 3 HALO study in patients with MO (*n* = 587) [18]. In a subgroup of patients with MO from the HALO study, fremanezumab resulted in significantly greater mean reductions from baseline in MMD at Month 3 with both quarterly and monthly dosing compared with placebo, with mean changes from baseline in MMD ranging from − 4.8 to − 5.2 at 3 months [18]. Given that secondary headache from MO is common among patients with migraine [29], these findings from both clinical trial and real-world settings support the benefits of fremanezumab treatment among patients with MO, which may be associated with more DTT migraine. Results from the current study indicate even greater reductions in MMD in patients with MO versus in clinical trials [18, 29], with a mean change from baseline in MMD of − 7.0 at 3 months, again reflecting the utility of fremanezumab in real-world practice.

Reductions in MMD and MHD with fremanezumab treatment for subgroups of patients with comorbid MDD or GAD were also reported in the current study, and improvements in the severity of these conditions were reported by approximately half of patients each with comorbid MDD or GAD. These results corroborate findings from a subgroup analysis of patients with comorbid moderate to severe depression (based on scores of ≥ 10 on the Patient the Health Questionnaire-9 [PHQ-9]; *n* = 229) from the phase 3 HALO study [30]. In that subgroup of patients with CM and comorbid moderate to severe depression, fremanezumab treatment resulted in significant reductions in MMD, as well as non-significant reductions in PHQ-9 scores, indicating a decrease in the severity of depressive symptoms [30]. Given the 5-fold higher risk for MDD and 3- to 5-fold higher risk for anxiety disorders among patients with migraine compared with healthy individuals [31–33], these findings, which showed improvement in migraine symptoms with fremanezumab in both real-world and clinical trial settings, as well as reductions in the severity of MDD and GAD with fremanezumab treatment in the current real-world study, show that fremanezumab may reduce the symptom burden of both migraine and these psychiatric comorbidities. Results from the current study versus the previous HALO CM study indicate slightly better outcomes for those with MDD (mean reductions from baseline in MMD, − 9.9 overall at 6 months versus − 6.5 with quarterly dosing and − 8.2 with monthly dosing at 6 months) [30]. This again supports fremanezumab as an effective treatment in clinical practice for patients with migraine and depression.

There has previously been a lack of evidence for the effectiveness of CGRP pathway–targeted mAbs in patients switching from another prior CGRP pathway–targeted mAb. Although effectiveness and reasons for switching were assessed in this newly explored and highly relevant subgroup of patients switching to fremanezumab from another prior CGRP mAb, there were several factors that were not taken into account, including the duration of prior CGRP pathway–targeted mAb treatment, which CGRP mAb had been used, or if there was any washout period between treatments. Among patients who received prior CGRP pathway–targeted mAbs, the most common reasons for switching from another CGRP pathway–targeted mAb to fremanezumab were inadequate response to treatment and side effects. During the post-index period, patients who had switched from a prior CGRP pathway–targeted mAb experienced substantial reductions in MMD with fremanezumab treatment during the 6-month post-index period. Only 12% of patients who received a prior CGRP pathway–targeted mAb discontinued fremanezumab treatment after the index dose, most often due to poor response. These results suggest that fremanezumab is effective and well tolerated in patients with migraine who had failed a different CGRP pathway–targeted mAb and therefore that treatment failure to 1 CGRP pathway–targeted mAb does not predict treatment failure to another. This finding supports previous evidence showing that patients who had prior failure of a CGRP pathway–targeted mAb treatment achieved a clinical benefit by switching to another CGRP pathway–targeted mAb [34–36]. In a previous retrospective cohort study by Overeem and colleagues that included 25 patients who did not respond to erenumab and switched to either galcanezumab or fremanezumab, MHD were significantly reduced by Month 3 after switching and approximately 12% of patients achieved a ≥ 50% reduction in MHD [36]. In the current study, in the subgroup switching to fremanezumab from another prior CGRP mAb, the proportion of patients achieving a ≥ 50% reduction in MMD at Month 3 (45.5%) was substantially higher; however, the patient characteristics in the previous cohort study by Overeem and colleagues differed from the current study, with many patients in that prior study reporting daily headache [36].

There are several strengths in the design of this study. The real-world setting of this online physician chart review closely reflects the true clinical landscape of migraine. The broad range of clinicians (e.g., neurologists, general practitioners, NPs, PAs, psychiatrists) included in this study may more accurately reflect real-world use of fremanezumab and enhance the generalizability of these results. In addition, the large patient sample size allowed for a number of subgroup analyses to be conducted, including the currently presented analyses in populations by migraine type or with DTT migraine (MO, MDD, GAD, or prior CGRP pathway–targeted mAb exposure). Furthermore, the inclusion of patients with a physician-confirmed migraine diagnosis and collection of both health care provider– and patient-reported outcomes have given a comprehensive view into the clinical outcomes across the broad population of patients with migraine. The results from this real-world study further support the effectiveness of fremanezumab as demonstrated in previous clinical trials [15–17], including in patients with DTT migraine [17–19, 30], and contribute to the real-world data on fremanezumab and other CGRP pathway–targeted mAbs [22, 24, 37–45].

This real-world study had limitations that need to be considered. Retrospective real-world studies are subject to bias and confounding factors, problems that are controlled for in randomized controlled trials. For the subgroups with MDD or GAD, the diagnosis was based only on the diagnosis noted in the chart, which was not required to be made by a qualified mental health professional and was not based on any specific definition or guidance in the current study. Thus, there is the potential that these diagnoses were not completely accurate for all patients included in those subgroups. Moreover, data collection efforts were limited by the availability of clinical data in the medical charts. With that said, observed discontinuation rates were relatively low (~ 8%), suggesting that discontinuation is not the cause of the limited availability of certain data. While measurements were conducted at monthly time points, it is unlikely that patients would be seen in the clinic every month, limiting availability of data at any individual month to those who came for follow-up in that month. Likewise, patients may have fewer follow-up visits if they experience a favorable therapeutic effect with fremanezumab and improvement in their symptoms. Other reasons may include physicians’ lack of reporting of the outcomes assessed at the follow-up appointments or the possibility that these follow-ups were not conducted in person. Although adherence to treatment, discontinuation rates, and reasons for discontinuation were reported over the full follow-up period, the length of this time frame varied among patients. As noted in the statistical analysis section, effectiveness outcomes clustered patients with available assessments within ± 15 days of each time point reported (e.g., Month 1, Month 2) due to heterogeneity in the assessment times observed in real-world practice. As a result, not all patients were included in the analyses for each time point and sample sizes varied accordingly. In addition, the mean follow-up period of 7 months was relatively short, with effectiveness outcomes evaluated over 6 months due to low follow-up numbers at later time points. Reporting rates declined over the course of the study; however, this decrease over time in the proportion of patients who responded to each outcome was not necessarily due to discontinuation of treatment, as described previously. In addition, certain outcomes that may have been of interest for some of the subgroups, including detoxification prior to initiating fremanezumab or the proportion of patients reverting from MO to no MO with fremanezumab treatment in the MO subgroup, were not collected in the eCRF. In line with real-world clinical practice, some patients in the current study were using concomitant preventive medications with fremanezumab, which could potentially have had an impact on observed effectiveness outcomes; however, preventive medication use decreased after fremanezumab initiation. MMD and MHD also decreased substantially after fremanezumab initiation, indicating that the addition of fremanezumab treatment led to improvements in both outcomes. Furthermore, approximately 20% of patients in the HALO CM pivotal study were using concomitant preventive medication [15]; efficacy, including reduction in MHD, was comparable in subgroups of patients from HALO CM with and without preventive medication use [46].

## Conclusions

Sustained reductions in MMD and MHD and clinically meaningful (≥ 50%) reductions in MMD were observed, with consistent effectiveness results across subgroups of patients with EM or CM and those with DTT migraine, including patients with acute MO, comorbid MDD, comorbid GAD, and prior exposure to another CGRP pathway–targeted mAb. Taken together, the results from this real-world study reinforce the effectiveness of fremanezumab as a migraine preventive treatment across the full spectrum of patients with migraine, including those with comorbidities or other factors that may contribute to DTT migraine.

## Supplementary Information

**Additional file 1.**

## Data Availability

All data for the analyses presented in this manuscript are included in this published article and its supplementary information files.

## Abbreviations

BL: Baseline
CGRP: Calcitonin gene-related peptide
CM: Chronic migraine
DTT: Difficult-to-treat
eCRF: Electronic case report form
EM: Episodic migraine
GAD: Generalized anxiety disorder
mAb: Monoclonal antibody
MDD: Major depressive disorder
MHD: Monthly headache days
MMD: Monthly migraine days
MO: Medication overuse
SD: Standard deviation
