# Supplementary material for: Real-world effectiveness after initiating fremanezumab treatment in US patients with episodic and chronic migraine or difficult-to-treat migraine
Source: J Headache Pain. 2022 May 16;23(1):56. doi: 10.1186/s10194-022-01415-x (PMC9109352; doi:10.1186/s10194-022-01415-x)
Supplement: Supplementary file 1 — Additional file 1. [file 10194_2022_1415_MOESM1_ESM.docx]

**Supplemental Table 1. Acute and Preventive Medications Taken Prior to and Concomitantly With Fremanezumab Treatment**

| **Medication, n (%)** | **Patients**  **with EM**  **(n = 416)** | | **Patients**  **with CM**  **(n = 587)** | | **Patients**  **with MO**  **(n = 220)** | | **Patients**  **with MDD**  **(n = 134)** | | **Patients**  **with GAD**  **(n = 120)** | | **Prior CGRP exposure**  **(n = 98)** | |
| --- | --- | --- | --- | --- | --- | --- | --- | --- | --- | --- | --- | --- |
|  | **Prior^a^** | **Comed^b^** | **Prior^a^** | **Comed^b^** | **Prior^a^** | **Comed^b^** | **Prior^a^** | **Comed^b^** | **Prior^a^** | **Comed^b^** | **Prior^a^** | **Comed^b^** |
| Preventive medications |  |  |  |  |  |  |  |  |  |  |  |  |
| Antiepileptics/  anticonvulsants | 241 (57.9) | 77 (18.5) | 413 (70.4) | 104 (17.7) | 164 (74.5) | 40  (18.2) | 100 (74.6) | 24 (17.9) | 91 (75.8) | 21  (17.5) | 67 (68.4) | 17  (17.3) |
| Antidepressants | 196 (47.1) | 63 (15.1) | 351 (59.8) | 122 (20.8) | 142 (64.5) | 54  (24.5) | 101 (75.4) | 51 (38.1) | 94 (78.3) | 39  (32.5) | 57 (58.2) | 18  (18.4) |
| Antihypertensives^c^ | 168 (40.4) | 33  (7.9) | 248 (42.2) | 40  (6.8) | 115 (52.3) | 20  (9.1) | 71 (53.0) | 13  (9.7) | 57 (47.5) | 9  (7.5) | 51 (52.0) | 10  (10.2) |
| Muscle relaxants | 95 (22.8) | 32  (7.7) | 185 (31.5) | 51  (8.7) | 100 (45.5) | 21  (9.5) | 59 (44.0) | 11  (8.2) | 55 (45.8) | 14  (11.7) | 40 (40.8) | 13  (13.3) |
| OnabotulinumtoxinA | 45 (10.8) | 6  (1.4) | 158 (26.9) | 41  (7.0) | 64 (29.1) | 14  (6.4) | 41 (30.6) | 5  (3.7) | 34 (28.3) | 6  (5.0) | 42 (42.9) | 5  (5.1) |
| Another anti-CGRP | 36  (8.7) | 5  (1.2) | 62 (10.6) | 8  (1.4) | 24 (10.9) | 4  (1.8) | 22 (16.4) | 2  (1.5) | 22 (18.3) | 4  (3.3) | 98 (100.0) | 6  (6.1) |
| Antihistamine | 23  (5.5) | 6  (1.4) | 47  (8.0) | 7  (1.2) | 27 (12.3) | 7  (3.2) | 20 (14.9) | 3  (2.2) | 15 (12.5) | 2  (1.7) | 15 (15.3) | 2  (2.0) |
| Acute medications |  |  |  |  |  |  |  |  |  |  |  |  |
| Anti-migraine analgesics | 298 (71.6) | 196 (47.1) | 426 (72.6) | 294 (50.1) | 178 (80.9) | 124 (56.4) | 111 (82.8) | 75  (56.0) | 98 (81.7) | 70  (58.3) | 68 (69.4) | 53  (54.1) |
| NSAIDs | 233 (56.0) | 144 (34.6) | 373 (63.5) | 243 (41.4) | 172 (78.2) | 100  (45.5) | 94 (70.1) | 61  (45.5) | 89 (74.2) | 63  (52.5) | 61 (62.2) | 42  (42.9) |
| Butalbital-containing compounds | 122 (29.3) | 27  (6.5) | 200 (34.1) | 37  (6.3) | 110 (50.0) | 25  (11.4) | 56 (41.8) | 6  (4.5) | 53 (44.2) | 6  (5.0) | 39 (39.8) | 6  (6.1) |
| Narcotics/opioids | 90 (21.6) | 12  (2.9) | 122 (20.8) | 20  (3.4) | 78 (35.5) | 11  (5.0) | 40 (29.9) | 4  (3.0) | 36 (30.0) | 2  (1.7) | 22 (22.4) | 6  (6.1) |
| Ditans | 52 (12.5) | 25  (6.0) | 45  (7.7) | 16  (2.7) | 30 (13.6) | 19  (8.6) | 13  (9.7) | 3  (2.2) | 10  (8.3) | 3  (2.5) | 14 (14.3) | 3  (3.1) |
| Other analgesics | 27  (6.5) | 5  (1.2) | 43  (7.3) | 10  (1.7) | 31 (14.1) | 6  (2.7) | 19 (14.2) | 3  (2.2) | 15 (12.5) | 7  (5.8) | 18 (18.4) | 5  (5.1) |
| Gepants | 31  (7.5) | 40  (9.6) | 33  (5.6) | 30  (5.1) | 25  (11.4) | 27  (12.3) | 8  (6.0) | 9  (6.7) | 8  (6.7) | 4  (3.3) | 14 (14.3) | 6  (6.1) |
| Other acute or preventive treatment | 0 | 0 | 7  (1.2) | 2  (0.3) | 2  (0.9) | 0 | 3  (2.2) | 1  (0.7) | 5  (4.2) | 2  (1.7) | 1  (1.0) | 1  (1.0) |
| No prior acute or preventive treatment/no concurrent treatment | 14  (3.4) | 53  (12.7) | 18  (3.1) | 80  (13.6) | 2  (0.9) | 17  (7.7) | 0 | 14  (10.4) | 1  (0.8) | 11  (9.2) | 0 | 16  (16.3) |

CGRP, calcitonin gene-related peptide; CM, chronic migraine; EM, episodic migraine; GAD, generalized anxiety disorder; MDD, major depressive disorder; MO, medication overuse.

^a^Medications taken in the 12 months prior to fremanezumab treatment initiation. ^b^Medications taken concomitantly with fremanezumab treatment.
